# Adverse events following immunization with DTaP-IPV (Tetraxim) in school-aged children in Taiwan, 2017–2020

**DOI:** 10.1016/j.jvacx.2024.100581

**Published:** 2024-11-07

**Authors:** Yen-Hsin Kung, Nan-Chang Chiu, Hsin Chi, Juan Camilo Vargas-Zambrano, Fu-Yuan Huang

**Affiliations:** a Department of Pediatrics, MacKay Children’s Hospital, Taipei, Taiwan; b Department of Medicine, MacKay Medical College, New Taipei City, Taiwan; c Medical Department, Sanofi Vaccines, Lyon, France

**Keywords:** DTaP-IPV, Adverse event, Passive surveillance system, National immunization program, Pre-school booster

## Abstract

Vaccination has been an effective method to prevent and control diphtheria, tetanus, pertussis and polio diseases in infancy and adults for years. To maintain the protective effect, a DTaP-IPV vaccine, Tetraxim, was introduced into Taiwan’s national immunization program for children at 5 years of age after primary series vaccination in infancy in October 2017 replacing a Tdap-IPV. To survey the safety of this vaccine, data between 01 October 2017 and 31 December 2020 from two surveillance systems, the Vaccine Adverse Events Reporting System (VAERS) and Vaccine Injury Compensation Program (VICP), were reviewed. We analysed patient’s demographics, symptoms, time of onset, and outcome. A total of 667,497 doses of DTaP-IPV vaccine were administered during the study period. We combined data from VAERS and VICP and deleted duplicate subjects and reports. There was a total of 59 subjects with AEs and the reporting rate of AEs was 8.8 subjects per 100,000 doses. The most common AEs were injection site erythema and swelling. AEs occurred with a median 1 day after vaccination (range 0–3 days). Among the 59 subjects, eight (13.6 %) with serious AEs were hospitalized. These serious AEs included injection site erythema, swelling or extensive limb swelling after vaccination and hospitalization might have been due to ELS that was misdiagnosed as cellulitis. The national passive surveillance data support the safety profile of Tetraxim as a school-entry booster in children at 5 years old in Taiwan.

## Introduction

Diphtheria, tetanus, pertussis and polio cause morbidity and mortality worldwide particularly in children. Vaccination is an effective method to prevent and control these diseases [1]. In Taiwan, the national immunization program (NIP) recommends diphtheria, tetanus, and acellular pertussis (DTaP) and inactivated polio (IPV) vaccines at 2, 4 and 6 months of age as primary vaccinations, with a first booster dose at 18 months and a second booster dose after 5 years of age [2]. The pentavalent combination vaccines (DTaP-IPV-Hib) were used in the primary series and the first booster. Tetanus, reduced dose diphtheria and acellular pertussis-inactivated polio (Tdap-IPV) vaccine was introduced as a booter at 5 years of age since 2012. Tdap-IPV vaccine was later changed to DTaP-IPV, with higher concentrations of diphtheria toxoid and pertussis antigens, in October 2017 [3]. This is more aligned with the practices in European countries [4] and the United States [5] with the rationale that higher doses of pertussis components for children aged 4–6 years may improve the booster effect of pertussis vaccination.

The DTaP-IPV vaccine used in Taiwan is Tetraxim, manufactured by Sanofi. It is indicated for immunization in infants and children against diphtheria, tetanus, pertussis and polio as primary or booster vaccination. Each 0.5 mL dose of the vaccine contains: diphtheria toxoid (≥30 IU), tetanus toxoid (≥40 IU), two purified antigens of *Bordetella pertussis* (25 mcg of pertussis toxoid and 25 mcg of filamentous hemagglutinin), inactivated poliovirus containing type 1 (Mahoney, D-antigen, 40 units), Type 2 (MEF1, D-antigen, 8 units) and Type 3 (Saukett, D-antigen, 32 units) and 0.3 mg of aluminium hydroxide as adjuvant [6]. The clinical development program of Tetraxim started in the mid 1980′s and was conducted in various regions and countries. The primary doses of this vaccine have been shown to be safe and immunogenic [7]. Furthermore, Tetraxim as a school-entry booster has shown to induce a strong immune responses to all antigens, regardless of the type of primary series vaccines [8].

Very common adverse events (AEs) following vaccination according to product information leaflet are loss of appetite, nervousness, irritability, abnormal crying, somnolence, headache, vomiting, myalgia, malaise, fever, injection-site erythema, pain and swelling. Since the introduction of Tetraxim into Taiwan’s NIP as school-entry booster in October 2017, over six hundred thousand doses have been administered. It is essential to monitor the safety of Tetraxim in a large number of subjects in order to detect very rare AEs.

This study calculated and analysed the type and frequency of AEs reported to the Taiwan National Adverse Drug Reaction System (VAERS) and Vaccine Injury Compensation Program (VICP) following immunization of Tetraxim as a school-entry booster to determine its safety.

## Methods

This was a retrospective database analysis based on two passive surveillance systems of vaccine AEs in Taiwan. This study was approved by the Research Ethic Committee of MacKay Children’s Hospital, IRB No. 21MMHIS160e.

### Taiwan vaccine adverse events reporting system (VAERS)

The Taiwan VAERS governed by the Taiwan Centers for Disease Control (CDC), is a national passive surveillance system to monitor AEs following vaccination [9]. Vaccine recipients, healthcare providers, medical institutions, pharmaceutical companies or vaccine manufacturers reported AEs following immunization online or by mails. The standardized reporting form includes demographic data of patient, vaccine type, and descriptions of AEs. The reporting system administrators access the characteristics and severity of AEs to evaluate whether any potential new and/or relevant safety information comes out.

### Taiwan Vaccine Injury Compensation Program (VICP)

The VICP fund was established by the Ministry of Health and Welfare in 1988 [10]. The fund enables individuals to claim compensations from their local health bureau in the event of death, disabilities, serious illnesses, or adverse reaction following vaccination. The VICP Working Group reviews the claims and assesses the causal relationship between the vaccine and the AEs. Data of VICP include personal information, vaccine type, symptoms, treatment, outcome and causality assessment.

### Case definitions, data collection and analysis

We used preferred terms and system organ class standardized by Medical Dictionary for Regulatory Activities (MedDRA) to describe AEs. Swelling extending at least to the elbow or knee and involving most part of the injected limb with a diameter >10 cm was defined as extensive limb swelling (ELS) in VICP. However, symptoms were only recorded and did not be judged whether they meet the definition of ELS in VAERS. Serious AEs following immunization were defined as life-threatening illness or resulting in death, permanent disability, congenital anomaly, need for hospitalization or prolongation of hospitalization, or other irreversible damages according to the Taiwan Act of Adverse Event Reporting definition [11]. We reviewed the reports of AEs received by VAERS and applied to VICP following introduction of Tetraxim into Taiwan’s NIP for school-entry booster (≥5 years) from 01 October 2017 to 31 December 2020. To detect and remove duplicates, we cross-referenced the reports of AEs received by VAERS and VICP for patient age, sex, concomitant vaccination, time of onset after vaccination, symptom, and outcome. A single vaccine recipient may report one or more AEs. The time to onset was defined as the number of days from vaccination (day 0) to the event onset date. We obtained the number of Tetraxim administrated doses from Taiwan CDC. The reporting rates were calculated as per 100,000 doses distributed.

## Results

### AEs reported to VAERS

From 01 October 2017 to 31 December 2020, a total of 667,497 doses of DTaP-IPV vaccine were administered. From the database of Taiwan National VAERS, there were 46 subjects reporting 56 AEs following vaccine administration, corresponding to a reporting rate of 6.9 subjects (or 8.4 AEs) per 100,000 doses. The mean age of the subjects was 5.9 years (range 5.0–7.0 years). Twenty-seven subjects were males (58.7 %). The most common AE was injection site erythema and swelling (67.9 %), followed by fever (16.1 %). The other AEs are listed in Table 1. AEs occurred with a median 0.5 day after vaccination (range 0–3 days). Among the 46 subjects, two (4.3 %) had erythema, swelling, local heat and pain at the injection site and were hospitalized thus being considered as serious events; all other subjects reported non-serious AEs. The reporting rate for serious AEs was 0.3 subjects per 100,000 doses administered. The length of the hospitalization stays for both subjects was 4 days. There was no report of death following immunization. All subjects recovered from the AEs after treatment. Only one subject among the 46 had another concurrently administered vaccine (Japanese encephalitis vaccine).

**Table 1:** Reported adverse events to VAERS after Tetraxim immunization.

Adverse events* (total 56 events)	No. (%)
Injection site erythema and injection site swelling	38 (67.9)
Fever	9 (16.1)
Rash pruritic	2 (3.6)
Fatigue	1 (1.8)
Decreased appetite	1 (1.8)
Headache	1 (1.8)
Dizziness	1 (1.8)
Strabismus	1 (1.8)
Tachycardia	1 (1.8)
Epistaxis	1 (1.8)

* Adverse events by preferred term name in Medical Dictionary for Regulatory Activities (MedDRA) coding.

### AEs reported to VICP

During the same period, Taiwan VICP had 19 subjects reporting 19 AEs following DTaP-IPV vaccination. However, two AEs were assessed as not related to vaccination by the review committee of VICP therefore excluded from further analysis. There were 1, 7, 6 and 3 AEs in the years of 2017, 2018, 2019 and 2020, respectively. Fourteen subjects were males (14/17, 82.4 %). AEs occurred at a median of 1 day after vaccination (range 0–2 days). DTaP-IPV was the only vaccine administered in 14 (82.4 %) of the 17 subjects. The concurrently administered vaccines in the remaining three subjects were measles, mumps, rubella (MMR), influenza, and MMR plus varicella, respectively. There were 9 non-serious cases and 8 serious cases. Among the 17 AEs, 4 (23.5 %) were injection site erythema and swelling and the remaining 13 (76.5 %) were ELS. Eight (61.5 %) subjects that experienced ELS were hospitalized. The median length of hospital stay was 3.5 days (range 1–4). There was no report of death following DTaP-IPV immunization. All subjects recovered completely.

### Combined data from VAERS and VICP

The database from VAERS and VICP were both de-identified. However, we deleted 4 duplicated subjects based on the demographic data (patient age, sex, date of injection and date of symptoms onset) and the medical history. There was a total of 59 subjects included. The reporting rate was 8.8 subjects per 100,000 doses (Table 2). The mean age of the subjects was 5.7 years (range 5.0–7.0 years). AEs occurred with a median of 1 day after vaccination (range 0–3 days). Thirty-seven subjects were males (62.7 %). Eight (13.6 %) subjects with serious AEs were hospitalized due to ELS. Non-serious cases included injection site erythema and injection site swelling, fever, fatigue, decreased appetite, pruritic rash, headache, dizziness, tachycardia and epistaxis.

**Table 2:** Reporting rate of AEs combined data from VAERS and VICP.

Timeframe	All administered doses	Subjects	Reporting rate (Subjects per 100,000)
2017 Oct.–Dec.	22,214	3	13.5
2018	212,399	30	14.1
2019	225,806	20	8.9
2020	207,078	6	2.9
2017 Oct.-2020	667,497	59	8.8

The reporting rate for serious AEs was 1.2 subjects per 100,000 doses administered. The median length of hospital stay was 3.5 days (range 1–4 days). All eight of the subjects who reported ELS were hospitalized under the suspicion of cellulitis and received antimicrobial treatment. The demographics of subjects reporting serious and non-serious AEs are shown in Table 3. Subjects reporting serious AEs and non-serious AEs appear similar in regard to age, sex, and time of AE onset.

**Table 3:** Demographics of the subjects reporting adverse events combined data from VAERS and VICP.

Variable	All (N = 59)	Serious (N = 8)	Non-serious (N = 51)
Age (years)	5.7	5.5	5.4
Male	37	6	31
Onset time, median days (range)	1 (0–3)	1 (0–3)	0 (0–2)

## Discussion

Pertussis remains a disease of concern in Taiwan, and worldwide as it is one of the most common vaccine-preventable diseases. The study based on cases reported to the Taiwan CDC from 2003 to 2017 demonstrated that infants had highest incidence rates of confirmed pertussis, followed by children aged 4 years, 5–9 years and 10–14 years [12], [13]. In a study conducted in northern Taiwan to evaluate the seroepidemiology of pertussis among elementary school children, only one-third of elementary school children were seropositive and seroprevalence declined with increasing class grades [14]. Despite high immunization coverage rates (>98 % for DTaP-IPV primary doses in Taiwan) [15], pertussis still remains a public health concern. Immunity acquired from primary series vaccination in school-age children is attenuated over time [16]. These children may become susceptible to pertussis and act as a source infection to young infants. Consequently, a Tdap-IPV booster was introduced in Taiwan in 2012, but changed to a DTaP-IPV booster in October 2017 with the rationale that a higher content of pertussis antigens would induce a better and longer-lasting immunity.

The safety of Tetraxim vaccine was assessed through multiple clinical trials and post-marketing studies in South Korea, all concluding that the vaccine is well-tolerated without posing specific concerns [6], [7], [17]. Nevertheless, a study of this type, relying on a large database with hundreds of thousands of doses distributed, had not been conducted before for Tetraxim. Taiwan’s national VAERS and VICP are both passive surveillance systems used to monitor AEs following immunization. The VAERS is mainly reported by healthcare workers, while parents can apply to the VICP free of charge. Reports of vaccine recipients who had mild AEs might be missed, but for those who have more severe AEs, parents should be willing to submit to VICP because no application fee is required. Furthermore, they could be eligible for a compensation fee if a causal association between the AE and the vaccine is demonstrated. While AEs reported to VAERS are not assessed for causality, the medical records of subjects applying to VICP are carefully reviewed to make a causality judgment. In the study period, two AEs were determined to be not related to vaccination by the committee of VICP, and the other 17 AEs were judged as being causally related to a vaccine. Due to the inherent limitations of such systems, the number of AEs reported might be underestimated especially those that are considered mild and expected by parents and physicians. Nonetheless more serious adverse events are expected to be reported and are important for pharmacovigilance purposes. This study poses a unique advantage as it could enrol as many cases with AEs as possible by using these two national surveillance systems in a real-world setting, particularly in a setting where a vaccine with a higher antigen content was replacing another with a lower antigen content. The main reporters of these two systems were different. As long as either system was notified, we could get the information, which would increase the probability to capture cases with AEs. Despite this study being the largest of its kind, including various years of exposure, it has limited power to catch events that happen with a frequency lower than 1 in a 500,000 doses.

In our study, local reactions (erythema and swelling) were the most frequent AEs, followed by fever. The post-marketing surveillance of DTaP-IPV as primary doses in infants and boosters in school-age children conducted in Korea with a duration of 6 years also revealed injection site reactions including tenderness/pain, erythema and swelling were the most frequently observed solicited reactions. Fever was also observed in 3.1 % of participants [6]. However, most AEs were of mild intensity, and all participants recovered. No safety concerns were observed related to the DTaP-IPV vaccination in the study.

ELS was the majority of serious AEs in the study and was determined to be vaccine-related by the VICP. Extensive swelling of a limb after a booster dose of acellular pertussis-containing vaccine has been documented for decades [8], [18]. ELS could also occur after other vaccine injections [19], [20]. Although the majority of serious AEs reported here were ELS, the reporting rates are lower than in other studies. Based on reviews of this topic, the frequency of ELS was about 2–6 % of children receiving a booster dose, which is significantly higher rates to those reported here [21], [22]. However, these studies had a smaller number of cases and analysed the reactions after vaccination using diary cards recorded by parents. Additionally, a larger retrospective study (32,887 DTaP vaccines given as 4th or 5th doses) was conducted relying on an administrative database in children vaccinated with a DTaP-vaccine (without IPV) in the United States between 1997 and 2000. This study showed 10 reports of swelling compromising at least 6 cm of the limb where the vaccine was given of which 5 were consistent with whole-limb involvement, and 3 specifically for those receiving the 5th dose. Those event numbers translate to the reporting rates of 15.2 per 100,000 doses for swelling more than >6 cm, and 18.5 per 100,000 doses for whole-limb involvement for the 5th dose [23]. The aforementioned study was a population-based surveillance that utilized a larger number of databases in children. The estimated incidence of ELS in that study was similar to our results reported here. In the case of this DTaP-IPV vaccine, ELS is already mentioned in the product monograph as a potential adverse event of unknown frequency.

The mechanism of extensive swelling in the injected limb is not clearly understood but is likely to be multifactorial. It is not unique to pertussis vaccines and has been described for other vaccines as mentioned in Woo et al. 2003 following an USA’s VAERS analysis [24]. The phenomenon of ELS is similar to angioedema observed by ultrasound examination [25]. The ELS has been observed after acellular pertussis vaccine injection but as mentioned before in a large study in United States. It was observed with many other vaccines and even recently with a mRNA COVID-19 vaccine. In pertussis vaccines, ELS occurs more often after booster dose (more likely after a 4 or 5th dose) than primary dose of vaccines and it might relative to cell-mediated immune response in older children [25], [26]. As well it was been suggested that prior sensitization to antigens or excipients contribute to ELS and not only, cellular immunity but as well humoral immunity plays a role. ELS might be misdiagnosed as bacterial cellulitis that tends to be accompanied with systemic fever and elevated C-reactive protein level (CRP) whereas the swelling in ELS usually resolves rapidly. According to data from VICP, 4 of 13 subjects with ELS had fever. We only had CRP data for 5 subjects. One of them was relatively high, 5.66 mg/dL, and the others were between 0.6–1.2 mg/dL. Based on previous study, patients with cellulitis had a mean CRP of 2.8 mg/dL [27]. Due to our limited data, while inflammatory markers such as CRP may be used to differentiate ELS from cellulitis when a patient presents with fever and localized extreme swelling, clinical judgment may be even more important. Misdiagnosis could lead to unnecessary diagnostic testing, antimicrobial treatment and even hospitalization. In the present study, the subjects who reported ELS were hospitalized for only a short period due to suspicion of cellulitis and were discharged recovered without any sequelae similar to what has been reported for most cases of ELS.

In conclusion, the national passive surveillance data support the safety profiles of Tetraxim as a school-entry booster in children at 5 years old in Taiwan. Our analysis demonstrates AEs following Tetraxim vaccination are very rare and mild. Local reactions, including ELS, were the most common AEs. Clinicians should have increased awareness of ELS to distinguish ELS from bacterial cellulitis and reduce unnecessary hospitalization and antimicrobial agent use.

## Author contributions

NC Chiu and JC Vargas-Zambrano contributed to conceptualization of the study. NC Chiu, YH Kung, H. Chi and FY Huang contributed to acquisition of data and data analysis and interpretation. NC Chiu, YH Kung and JC Vargas-Zambrano contributed to drafting of manuscript and critical revision. All authors attest that they meet the ICMJE criteria for authorship and have seen and approved the final version of the manuscript.

## CRediT authorship contribution statement

**Yen-Hsin Kung:** Writing – review & editing, Writing – original draft, Data curation. **Nan-Chang Chiu:** Writing – review & editing, Resources, Project administration, Methodology, Funding acquisition. **Hsin Chi:** Writing – review & editing, Resources. **Juan Camilo Vargas-Zambrano:** Writing – review & editing, Project administration, Methodology, Funding acquisition. **Fu-Yuan Huang:** Writing – review & editing, Resources.

## Funding

This study was funded by Sanofi. No.PER00099.

## Declaration of competing interest

The authors declare the following financial interests/personal relationships which may be considered as potential competing interests: Nan-Chang Chiu reports financial support was provided by Sanofi. Juan Camilo Vargas-Zambrano reports a relationship with Sanofi that includes: employment and owning shares.

## Data Availability

Data will be made available on request.

## References

[1] WHO. Immunization and vaccine-preventable communicable diseases. WHO website, https://www.who.int/data/gho/data/themes/immunization [accessed 1 May 2023].

[2] Taiwan CDC. Time schedule of vaccinations. Taiwan CDC website, https://www.cdc.gov.tw/Category/Page/TxRW-x3WzvPhvEtxM628GA [accessed 1 May 2023].

[3] Taiwan CDC press release Oct 10 2017. Taiwan CDC website, https://www.cdc.gov.tw/Category/ListContent/4T8vDeSxOpmz0ILvF_D8Yw?uaid=40MxK_3K72ygwh1LLCKRBg [accessed 1 May 2023].

[4] European Centre for Disease Prevention and Control. Vaccine schedules in all countries in the EU/EEA, https://vaccine- schedule.ecdc.europa.eu/ [accessed 1 May 2023].

[5] Liang JL, Tiwari T, Moro P, Messonnier NE, Reingold A, Sawyer M, et al. Prevention of Pertussis, Tetanus, and Diphtheria with Vaccines in the United States: Recommendations of the Advisory Committee on Immunization Practices (ACIP). MMWR Recomm. Rep. 8 Apr 27;67(2):1-44. doi: 10.15585/mmwr.rr6702a1. 10.15585/mmwr.rr6702a1 PMC5919600 29702631

[6] Choe Y.J., Vidor E., Manson C. (2022). Post-marketing surveillance of tetravalent diphtheria-tetanus-acellular pertussis and inactivated poliovirus (DTaP-IPV) vaccine in South Korea, 2009 to 2015. Infect Dis Ther.

[7] Lee S.Y., Hwang H.S., Kim J.H., Kim H.H., Lee H.S., Chung E.H. (2011). Immunogenicity and safety of a combined diphtheria, tetanus, acellular pertussis, and inactivated poliovirus vaccine (DTaP-IPV) compared to separate administration of standalone DTaP and IPV vaccines: a randomized, controlled study in infants in the Republic of Korea. Vaccine.

[8] Huoi C., Vargas-Zambrano J., Macina D., Vidor E. (2022). A combined DTaP-IPV vaccine (Tetraxim®/Tetravac®) used as school-entry booster: a review of more than 20 years of clinical and post-marketing experience. Expert Rev Vaccines.

[9] Taiwan CDC. VAERS. https://www.cdc.gov.tw/Category/Page/3-aXlTBq4ggn5Hg2dveHBg [accessed 1 May 2023].

[10] Taiwan CDC. Vaccine Injury Compensation Program (VICP) https://www.cdc.gov.tw/Uploads/archives/4af45ac1-6183-4567-9b5b-eaf625685048.pdf [accessed 1 May 2023].

[11] Taiwan FDA. Taiwan Act of Adverse Event Reporting definition. http://www.fda.gov.tw/tc/siteContent.aspx?sid=4240 [accessed 1 May 2023].

[12] Chang I.F., Lee P.I., Lu C.Y., Chen J.M., Huang L.M., Chang L.Y. (2019). Resurgence of pertussis in Taiwan during 2009–2015 and its impact on infants. J Microbiol Immunol Infect.

[13] Macina D., Evans K.E. (2021). Bordetella pertussis in school-age children, adolescents, and adults: A systematic review of epidemiology, burden, and mortality in Asia. Infect Dis Ther.

[14] Kuo C.C., Huang Y.C., Hsieh Y.C., Huang Y.L., Huang Y.C., Hung Y.T. (2017). Seroepidemiology of pertussis among elementary school children in northern Taiwan. J Microbiol Immunol Infect.

[15] Taiwan CDC. Immunization coverage rates. https://www.cdc.gov.tw/Category/MPage/S2UF2-VuMgfzgzpy7qdvlA [accessed 1 May 2023].

[16] Gao H., Lau E.H.Y., Cowling B.J. (2022). Waning immunity after receipt of pertussis, diphtheria, tetanus, and polio-related vaccines: a systematic review and meta-analysis. J Infect Dis.

[17] Pancharoen C., Chotpitayasunondh T., Chuenkitmongkol S., Ortiz E. (2012). Long-term immunogenicity assessment of a DTaP-IPV//PRP-T vaccine given at 2, 4, 6 and 18–19 months of age, and immunogenicity and safety of a DTaP-IPV vaccine given as a booster dose at 4 to 6 years of age in Thai children. Southeast Asian J Trop Med Public Health.

[18] Southern J., Waight P.A., Andrews N., Miller E. (2017). Extensive swelling of the limb and systemic symptoms after a fourth dose of acellular pertussis containing vaccines in England in children aged 3–6years. Vaccine.

[19] Cohen E., Tahri S., Lukkassen I. (2015). Two cases of extensive limb swelling after influenza vaccination. Pediatr Infect Dis J.

[20] Recher M., Hirsiger J.R., Bigler M.B., Iff M., Lemaître B., Scherer K. (2018). Immune system correlates of extensive limb swelling in response to conjugated pneumococcal vaccination. npj Vaccines.

[21] Huber B.M., Goetschel P. (2011). Extensive limb swelling after vaccination. J Pediatr.

[22] Rennels M.B. (2003). Extensive swelling reactions occurring after booster doses of diphtheria-tetanus-acellular pertussis vaccines. Semin Pediatr Infect Dis.

[23] Jackson L.A., Carste B.A., Malais D., Froeschle J. (2002). Retrospective population-based assessment of medically attended injection site reactions, seizures, allergic responses and febrile episodes after acellular pertussis vaccine combined with diphtheria and tetanus toxoids. Pediatr Infect Dis J.

[24] Woo E.J., Burwen D.R., Gatumu S.N.M., Ball R. (2003). Vaccine adverse event reporting system working group. Extensive limb swelling after immunization: reports to the vaccine adverse event reporting system. Clin Infect Dis.

[25] Marshall H.S., Gold M.S., Gent R., Quinn P.J., Piotto L., Clarke M.F. (2006). Ultrasound examination of extensive limb swelling reactions after diphtheria-tetanus-acellular pertussis or reduced-antigen content diphtheria-tetanus-acellular pertussis immunization in preschool-aged children. Pediatrics.

[26] Scheifele D.W., Halperin S.A., Ferguson A.C. (2001). Assessment of injection site reactions to an acellular pertussis-based combination vaccine, including novel use of skin tests with vaccine antigens. Vaccine.

[27] Harper B.D., Marcus C.H., Burke N., Kawai K., Mansbach J.M. (2021). Utility of inflammatory markers in hospitalized children with skin erythema. Hosp Pediatr.

